# Gaps of Social Support Among Elderly Chinese Residents in Independent Living Facilities - an Intercultural Communication Thematic Analysis

**DOI:** 10.1017/ash.2025.383

**Published:** 2025-09-24

**Authors:** Cheyu Zhang, Jhumka Gupta, Rochelle Davidson Mhonde

**Affiliations:** 1 George Mason University

## Abstract

**Background:** Elderly Chinese residents who live in independent living facilities are a fast-growing but under-researched population. For instance, there is a lack of research regarding the various forms of social support that Chinese residents need and the barriers to receiving social support that impact health outcomes for Chinese residents. In addition, COVID-19 adds another layer of stress to Chinese people with increased social stigma. This study aims to investigate the experiences of elderly Chinese residents in an independent living facility and the existing social support gaps. **Method:** 16 residents from one independent living facility were recruited. Semi-structured interviews were conducted to understand their social support needs and the gaps they experienced. Interviews were conducted in Chinese with an interviewer fluent in Chinese and English. We analyzed the data using thematic analysis based on the stress, coping, and social support gaps frameworks, which resulted in twenty-seven codes, eight subthemes, and three major themes. **Results:** The three main themes include: (1) Stressors based on experiences and intercultural communication challenges between healthcare providers, facility staff, and residents. Most residents are aware of their aging and risk of death through experienced or observed adverse events, including stigma and events associated with COVID-19. Communicational, cultural, and instrumental challenges during the coping and help-seeking process intensify chronic stressors in the residents’ daily lives. (2) Social support received by the residents that helps with their coping. Residents receive some level of emotional and instrumental support, with children’s support being the most complete and concrete source of support. Peer support and facility support form layers of protection for residents to maintain their health. (3) Social support gaps between residents’ needs and their received social support and associated psychological symptoms. Many residents experience helplessness and dependence on their children with their current social support experience. Passiveness in help-seeking when facing stressors is also observed. Implications: Independent living facilities with Chinese residents must ensure that their providers’ communication is culturally responsive. In addition, facilities should also prioritize communicating with family members to provide social support to Chinese residents to cope with challenges in their lives and improve their physical and mental health.

